# Supplementary material for: Impact of pole dancing on mental wellbeing and sexual self-concept: Protocol for a systematic review and meta-analysis
Source: PLoS One. 2024 Jul 15;19(7):e0307182. doi: 10.1371/journal.pone.0307182 (PMC11249261; doi:10.1371/journal.pone.0307182)
Supplement: Attachment — Submitted filename: Response to Reviewers.doc [file pone.0307182.s002.doc]

Dear Academic Editor:

Thanks for providing us with this great opportunity to submit a revised version of our manuscript. We appreciate the detailed and constructive comments provided by you. We have carefully revised the manuscript by incorporating all the suggestions. The modified parts of the paper are recolored in red.

1. “Please ensure that your manuscript meets PLOS ONE's style requirements, including those for file naming.”

We have recalibrated the format to conform to PLOS ONE's style requirements.

2. “We note you have included a table to which you do not refer in the text of your manuscript. Please ensure that you refer to Table 1 in your text; if accepted, production will need this reference to link the reader to the Table.”

We have added the following to the article. “Please refer to Table 1 for details.”For details, please see lines 118-119.

3. “We note that this manuscript is a systematic review or meta-analysis; our author guidelines therefore require that you use PRISMA guidance to help improve reporting quality of this type of study. Please upload copies of the completed PRISMA checklist as Supporting Information with a file name ‘PRISMA checklist’.”

Thank you very much for your reminding. We have already uploaded this checklist as Supporting Information with a file name ‘PRISMA checklist’.

In addition, we have added the following to the abstract .

“This systematic review was registered in the PROSPERO. We will follow the Preferred Reporting Items for Systematic Reviews and Meta-analysis Protocol to accomplish the systematic review protocol. “

4. “Please review your reference list to ensure that it is complete and correct. If you have cited papers that have been retracted, please include the rationale for doing so in the manuscript text, or remove these references and replace them with relevant current references. Any changes to the reference list should be mentioned in the rebuttal letter that accompanies your revised manuscript. If you need to cite a retracted article, indicate the article’s retracted status in the References list and also include a citation and full reference for the retraction notice.”

We have rechecked the reference list to ensure that it is complete and correct.

Thank you again for your valuable advice. We have further refined our protocol and made the upcoming studies more standardized.

Dear Reviewer #1:

Thank you very much for your careful review! We have carefully studied your valuable comments and have made amendments one by one. We hope that these adjustments will meet your requirements!

1. *“The introduction is long and tiring. The suggestion for the authors is to summarize the content featuring the main theme, pole dancing, followed by text with the most up-to-date references of studies that show potential psychological benefits, maintaining the important gap of there being no systematic reviews specifically on pole dancing and concluding the introduction with the main objective.”*

We have streamlined the introduction. We cut out a paragraph. The paragraph reads as follows:

“Additionally, dance is critical in augmenting bodily awareness, fostering mental health, and facilitating neurorehabilitation(7). The release of endorphins triggered by dance activities has been linked to significant improvements in mental well-being(8). In essence, dance offers a holistic therapeutic avenue for enhancing mental health, embodying various psychological benefits such as improved self-esteem, stress mitigation, emotional health, and heightened bodily consciousness. This extensive body of research underscores dance's therapeutic potential in advancing mental health and supporting psychological well-being across various populations. ”

In addition, we have removed the following from the introduction:

“The multifaceted nature of dance, characterized by its rhythm, musicality, social engagement, technical and physical demands, and capacity to facilitate connection, mindfulness, and the expression of aesthetic emotions, contributes to its overall health and well-being benefits.(9)”

“The intersectionality of pole dancing with feminist theory further highlights its role in dialogues concerning gender, sexuality, and empowerment(13). ”

2. *Finally, the methodology is well constructed, but the text "Our primary outcomes of interest are the degrees of mental well-being and the enhancement or alteration of the sexual self-concept.", does not provide the specificity required in a SR and should be divided. Authors should consider the degrees of mental well-being as a primary outcome, citing validated scales that need to have been used in RCTs to be included in the SR (Ex: The Warwick-Edinburgh Mental Well-being Scale (WEMWBS), Ryff Psychological Well -being (PWBS-42) scale). In this way, they would consider enhancement and alteration of the sexual self-concept as secondary outcomes, also including validated scales that should be present in RCTs, such as The Multidimensional Sexual Self-Concept Questionnaire (MSSCQ).*

We adjusted part of the article and identified mental wellbeing and sexual self-concept as the primary outcome and secondary outcome respectively. Finally, we have included in the article specific scales used in the measurement process. For details, please see lines 134-140.

3. The inclusion in the methodology of the PICOS strategy (P = Adults, I = pole dancing, C = usual activity, or other type of physical activity/dance, O = mental well-being, sexual self-concept assessed by the scales, S = RCT) will further enrich this section.

We have adopted the "PICOS" principle to present the inclusion criteria according to your suggestion.For details, please see lines 126-143.

Thank you again for your valuable advice. We have further refined our protocol and made the upcoming studies more standardized.
